# Identifying chondrogenesis strategies for tissue engineering of articular cartilage

**DOI:** 10.1177/2041731419842431

**Published:** 2019-04-22

**Authors:** Michael J Chen, Jonathan P Whiteley, Colin P Please, Franziska Ehlicke, Sarah L Waters, Helen M Byrne

**Affiliations:** 1 School of Mathematical Sciences, The University of Adelaide, Adelaide, SA, Australia; 2 Mathematical Institute, University of Oxford, Oxford, UK; 3 Department of Computer Science, University of Oxford, Oxford, UK; 4 Department of Tissue Engineering and Regenerative Medicine, University Hospital Würzburg, Würzburg, Germany

**Keywords:** Tissue engineering, chondrogenesis, TGF-*β*, reaction-diffusion model

## Abstract

A key step in the tissue engineering of articular cartilage is the chondrogenic differentiation of mesenchymal stem cells (MSCs) into chondrocytes (native cartilage cells). Chondrogenesis is regulated by transforming growth factor-*β* (TGF-*β*), a short-lived cytokine whose effect is prolonged by storage in the extracellular matrix. Tissue engineering applications aim to maximise the yield of differentiated MSCs. Recent experiments involve seeding a hydrogel construct with a layer of MSCs lying below a layer of chondrocytes, stimulating the seeded cells in the construct from above with exogenous TGF-*β* and then culturing it in vitro. To investigate the efficacy of this strategy, we develop a mathematical model to describe the interactions between MSCs, chondrocytes and TGF-*β*. Using this model, we investigate the effect of varying the initial concentration of TGF-*β*, the initial densities of the MSCs and chondrocytes, and the relative depths of the two layers on the long-time composition of the tissue construct.

## Introduction

**Figure 1.**
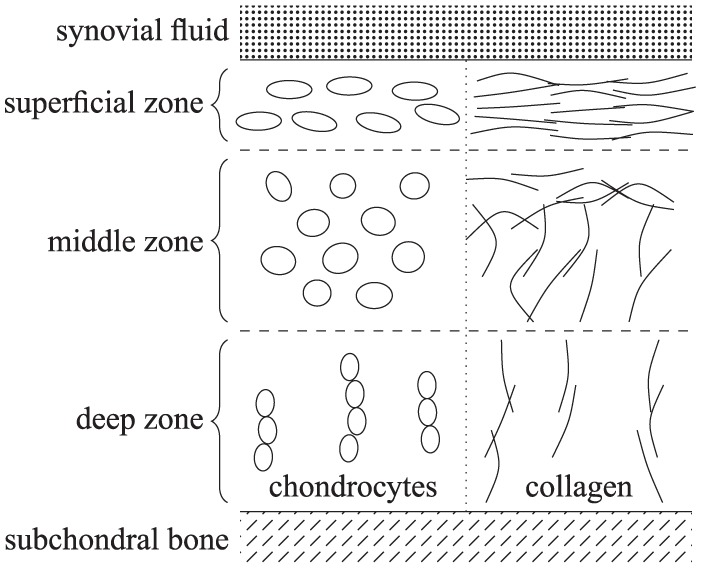
Schematic diagram of the zonated, depth-dependent structure of articular cartilage.

Articular cartilage acts as a lubricating surface in synovial joints, preventing bone contact. This thin tissue layer is composed of a dense network of extracellular matrix (ECM) components (principally collagen and proteoglycans), which is maintained by native cartilage cells, known as chondrocytes.^1^ As shown in Figure 1, the orientation and density of collagen varies with cartilage depth. In a synovial joint, the cartilage lies between the synovial fluid and the subchondral bone, and is typically described as comprising three zones. The highest density of collagen and chondrocytes is in the superficial zone, where both are aligned in the direction of the surface of the cartilage. In the middle zone, the cells are more rounded and the collagen alignment is less organised, with the density of both lower than in the superficial zone. In the deep zone, the cells are organised in column-like structures, with both these columns and the collagen aligned perpendicular to the subchondral bone.^1^ The zonated structure and interaction between its components endow cartilage with the mechanical properties necessary to withstand the high stress environment of a synovial joint.

Articular cartilage is an avascular tissue. This property, combined with the low proliferation and motility of the chondrocytes, means that it has a low capacity to regenerate when it degrades or is damaged through injury. Consequently, chronic conditions such as osteoarthritis require surgical intervention, typically involving a full joint replacement in end-stage disease. An approach which involves replacing damaged tissue with artificially engineered cartilage implants would be an attractive alternative to these invasive surgical procedures.

Artificially engineered cartilage implants aim to mimic the function of natural tissue. From a biomechanical perspective, they will ideally have a distribution of cells and matrix components similar to that shown in Figure 1.

A recent tissue engineering approach to develop such an implant^2^ has exploited additive manufacturing techniques to seed cells within a hydrogel construct reinforced with a three-dimensional (3D)-printed lattice of polymer fibres. The seeded cell population consists of mesenchymal stem cells (MSCs) and/or chondrocytes which are then biochemically and/or mechanically stimulated to promote chondrogenic differentiation of the MSCs and enhance matrix deposition by the chondrocytes. Both processes are regulated by transforming growth factor-*β* (TGF-*β*).^1^ Understanding how this chemokine drives MSCs to differentiate into chondrocytes as it diffuses through a tissue engineering construct is the focus of the present study.

**Figure 2.**
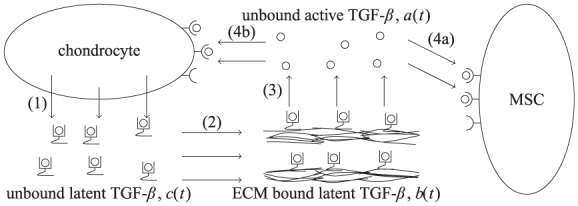
A schematic showing the simplified life cycle of TGF-*β* used in the model. The steps in this life cycle are (1) secretion of latent TGF-*β* by chondrocytes, (2) binding of latent TGF-*β* to the extracellular matrix, (3) activation (and release) of bound TGF-*β* by interaction with an activating chemical species and (4a, b) binding of active TGF-*β* to the receptors on MSCs and chondrocytes.

TGF-*β* plays an important role in both natural cartilage and in approaches to engineer artificial cartilage. It is produced in vivo by chondrocytes and binds rapidly to the ECM for storage. After undergoing an activation process, an active form of TGF-*β* is released from this stored state and can freely diffuse. It then acts to stimulate chondrocytes to synthesise ECM components, including collagen type II and proteoglycans (particularly aggrecan). Importantly for tissue engineering applications, it also drives MSCs to differentiate into chondrocytes. There are a number of subtleties to the biochemistry of TGF-*β* (as shown in Figure 2). TGF-*β* is endogenously secreted as part of a large molecular complex, this latent form comprises a ligand (the active component) bound to a protein and a peptide. As a result, the active component is unable to bind to receptors on the outer membrane of cells (in this study, MSCs and chondrocytes) until it is activated by being cleaved from this complex.

When first secreted, the latent TGF-*β* may freely diffuse, an arrangement (as part of a larger complex) which enables latent TGF-*β* to bind to and thus be stored throughout the ECM. Activation (i.e. the cleaving of just the active ligand from the complex) then occurs when chondrocyte stimulation is required (in response to ECM damage, for instance). TGF-*β* can be activated via mechanical interaction with cell integrins^3^ or via chemical interactions with proteases, thrombospondin-1, reactive oxygen species or extremes in pH.^4^ Once activated, it can freely diffuse but has a short half-life of only a few minutes, as compared to the timescale of days over which cell differentiation occurs.^5^ As a consequence, active TGF-*β* must bind rapidly to cell receptors to have an effect on the cells. In particular, if TGF-*β* binds to surface receptors of MSCs in sufficient quantity, they are driven to chondrogenic differentiation.^1^

For tissue engineering applications, there are two dominant sources of TGF-*β*: it may be secreted by chondrocytes or it may be added exogenously, where exogenous supplies may be replenished regularly to ensure a sustained effect on the cells. The key modelling question that we address in this article is to understand how these two sources of TGF-*β* act, either alone or in combination with each other, to direct stem cell differentiation. In particular, we seek to establish whether synergistic effects are possible where such a combination is more effective than relying solely on stimulation from either the TGF-*β* produced by the seeded chondrocytes or exogenous TGF-*β*.

In a previous study,^6^ we modelled chondrogenesis in well-mixed cell populations for which the distribution of the various forms of TGF-*β* were assumed to be spatially uniform. In that study, attention focussed on scenarios for which MSCs could be induced to differentiate into chondrocytes either by the addition of exogenous TGF-*β* or by co-culturing them with harvested chondrocytes. Analysis of an ordinary differential equation model representing these scenarios predicted that differentiation of the entire MSC population is induced if either sufficient concentrations of TGF-*β* is added or if the MSCs are co-cultured with a sufficiently high density of chondrocytes which themselves secrete TGF-*β*. Further simulations revealed and suggested synergistic effects between the two scenarios, so that experimental efficiencies could be achieved by adding exogenous TGF-*β* to a MSC/chondrocyte co-culture, as compared to using either strategy in isolation.

**Figure 3.**
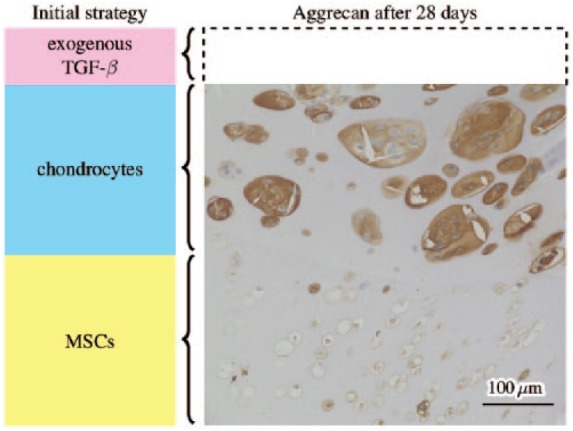
A schematic diagram of a layered MSC/chondrocyte co-culture stimulated with exogenous TGF-*β*, along with a microscope image from an experiment to develop a regenerative articular cartilage implant performed at University Hospital Würzburg.

The current work is motivated by recent experimental efforts to develop regenerative articular cartilage implants using a combination of additive manufacturing techniques and purpose-designed biomaterials.^2^ This includes experiments involving tissue engineering constructs where the initial seeded populations of MSCs and chondrocytes are spatially separated, see Figure 3. These constructs are comprised of cells embedded in a disc of PCL-reinforced hydrogel,^7^ and cultured in a bioreactor for a period of 28 days to promote the chondrogenic differentiation of the MSCs and the production of ECM. While in the bioreactor, the cell-seeded hydrogel disc is either free standing or housed in a sample of porcine tissue to mimic being implanted in a joint. Throughout this period, the construct is bathed in culture medium which contains exogenously added TGF-*β* replenished at regular intervals.

This layered construct was devised to mimic natural articular cartilage. The upper layer of chondrocytes correspond to the superficial zone of the cartilage, while the MSCs in the lower layer, once differentiated, are intended both to mimic its deep zone and to integrate with the subchondral bone (since MSCs may also be driven to an osteogenic rather than chondrogenic lineage). These initial experiments demonstrate that the constructs are biocompatible with the seeded cells. Furthermore, ECM components associated with cartilage are deposited in the upper, chondrocyte-seeded layer (the brown staining in Figure 3 is for aggrecan). These ECM components are not present in the lower layer, which suggests that chondrogenic differentiation of the MSCs had not occurred. Understanding these results motivates the present in silico study.

A schematic diagram of an idealised version of the cell seeding strategy used in these experiments is shown in Figure 3, with a layer of chondrocytes lying above a layer of MSCs and TGF-*β* stimulation coming above. This spatially simplified situation corresponds to only the central most part of the hydrogel disc, where stimulation from the sides of the construct may be neglected. Under this assumption, we only consider one spatial dimension in our mathematical model (parallel to the depth of the construct) and so focus our investigation on the effect of stimulating this central region from above.

**Figure 4.**
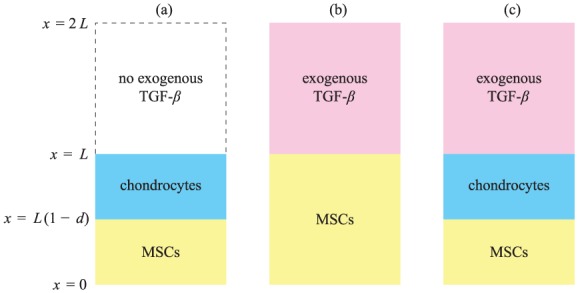
Schematic diagram of the three cell seeding and/or TGF-*β* stimulation strategies considered in this study: (a) Case study 1, (b) Case study 2 and (c) Case study 3.

We will consider a variety of strategies that combine zonated cell seeding and the addition of exogenous TGF-*β*, and determine the efficacy of each strategy to differentiate the seeded MSCs. The three seeding strategies under consideration and shown schematically in Figure 4, are

(1) layered MSC (lower) and chondrocyte (upper) cell seeding without exogenous TGF-*β*
(2) pure MSC cell seeding with exogenous TGF-*β*
(3) layered MSC (lower) and chondrocyte (upper) cell seeding with exogenous TGF-*β*

These strategies are analogous to the strategies considered in our previous model,^6^ with the addition of spatial dependence. Here, case study 3 corresponds to the experiments described above, where there was no observed lower layer differentiation, and so we aim to use our modelling approach to devise improved protocols for future in vitro studies. The goal is to make the most efficient use of the available chondrocytes, since these are in limited supply, and to use exogenous TGF-*β*, which is expensive, at as low a concentration as possible while still achieving chondrogenesis.

## Methods

### Mathematical model and background

We develop a mechanistic mathematical model to investigate how the spatial distributions of populations of MSCs and chondrocytes embedded in a layered hydrogel evolve over time in response to diffusion of TGF-*β*. We neglect cell motility since the cells are embedded in a dense hydrogel and are not observed to move. We assume that the chondrocytes neither proliferate nor dedifferentiate; these are reasonable assumptions for chondrocytes that have been derived from articular cartilage and that are being cultured in vitro.^8–10^ Over the timescale of interest (approximately 1–10 days), it is also reasonable to neglect cell death.^10^ For simplicity, we consider cell populations and TGF-*β* concentrations which vary with one spatial dimension x, that is parallel to the direction of the depth of the scaffold (see Figure 4).

The variables used to represent the various cell densities and forms of TGF-*β* are summarised in Table 1. We consider two distinct cell populations; we denote by m(x,t) and n(x,t) the density (number of cells/volume) of MSCs and chondrocytes, respectively, at depth x and time t. We assume that MSCs differentiate into chondrocytes if the amount of TGF-*β* bound to their receptors, denoted by f(x,t) (fraction of bound receptors/cell), exceeds the threshold value $f_{d}$. We apply the principle of mass balance to the two cell types to obtain the following equations for the evolution of m(x,t) and n(x,t)

(1) $$\mathrm{MSCs}:\;\frac{\partial m}{\partial t}=\underset{\mathrm{differentiation}\;\mathrm{of}\;\mathrm{MSCs}\;\mathrm{to}\;\mathrm{chondrocytes}}{\underset{︸}{-\Lambda_{1}H\left(f-f_{d}\right)m}}$$

(2) $$\mathrm{chondrocytes}:\;\frac{\partial n}{\partial t}=\underset{\mathrm{differentiation}\;\mathrm{of}\;\mathrm{MSCs}\;\mathrm{to}\;\mathrm{chondrocytes}}{\underset{︸}{\Lambda_{1}H\left(f-f_{d}\right)m}}$$

**Table 1.** Description of the model variables, along with their units.

Dependent variable	Description	Unit
m	Density of MSCs	Mio/mL
n	Density of chondrocytes	Mio/mL
c	Concentration of unbound, latent TGF-*β*	ng/mL
b	Concentration of bound, latent TGF-*β*	ng/mL
a	Concentration of unbound, active TGF-*β*	ng/mL
f	Fraction of bound TGF-*β* receptors per MSC	–
g	Fraction of bound TGF-*β* receptors per chondrocyte	–

MSC: mesenchymal stem cell; TGF-*β*: transforming growth factor-*β.*

‘Mio’ is an abbreviation of ‘million’.

In equations (1) and (2), H(⋅) is the Heaviside step function (H(z)=1 if z>0; H(z)=0 otherwise), so that differentiation is active when $f>f_{d}$. The use of a step function here is a biologically appropriate description of this process; see Ward and King^11^ for a comparison of step functions and Michaelis–Menten kinetics with a cell response that varies continuously with the amount of a receptor bound chemokine. In equations (1) and (2), the parameter $\Lambda_{1}$ represents the rate at which MSCs differentiate when $f>f_{d}$; a representative value of this parameter is given in Table 2, along with descriptions and representative values of all the model parameters that follow in equations (3)–(7).

**Table 2.** Summary of the dimensional parameters that appear in equations (1)–(7), together with estimates of their values.

Quantity	Description	Representative value
$\Lambda_{1}$	MSC to chondrocyte differentiation rate	$6\times 10^{-3}\;/\min$
$f_{d}$	MSC to chondrocyte differentiation threshold	0.01^6^
$\lambda_{3}$	Rate of constitutive TGF-*β* production by chondrocytes	$1.094\times 10^{-1}\mathrm{ng}/(\min\;\mathrm{Mio})$ ^8^
$\lambda_{4}$	Transfer rate between bound and unbound latent TGF-*β*	$10^{-1}/\min$ ^12^
$\lambda_{5}$	Decay rate of unbound latent TGF-*β*	$6\times 10^{-3}/\min$ ^13^
$\lambda_{6}$	Activation rate due to chemical interactions	$6\times 10^{-2}/\min$ ^8^
$\lambda_{7}$	Decay rate of bound latent TGF-β	$6\times 10^{-3}/\min$ ^13^
$\lambda_{8}$	Decay rate of active TGF-*β*	0.258/min ^13^
$\lambda_{m9}$	Binding rate of active TGF-*β* to MSC receptors	$6\times 10^{-2}/(\min\;\mathrm{ng}/\mathrm{mL})$ ^14^
$\lambda_{n9}$	Binding rate of active TGF-*β* to chondrocyte receptors	$6\times 10^{-2}/(\min\;\mathrm{ng}/\mathrm{mL})$ ^14^
$\lambda_{m10}$	Internalisation rate of TGF-*β* bound MSC receptors	0.25/min ^15^
$\lambda_{n10}$	Internalisation rate of TGF-*β* bound chondrocyte receptors	0.25/min ^15^
$F_{\mathrm{tot}}$	Maximum receptor bound TGF-*β* per MSC	$6.23\times 10^{-1}\mathrm{ng}/\mathrm{Mio}$ ^16^
$G_{\mathrm{tot}}$	Maximum receptor bound TGF-*β* per chondrocyte	$6.23\times 10^{-1}\mathrm{ng}/\mathrm{Mio}$ ^16^
$D_{c}$	Diffusivity of unbound latent TGF-*β*	$1.278\times 10^{-9}\;m^{2}/\min$ ^17^
$D_{a}$	Diffusivity of unbound active TGF-*β*	$1.278\times 10^{-9}m^{2}/\min$ ^17^

MSC: mesenchymal stem cell; TGF-*β*: transforming growth factor-*β.*

As shown in Figure 2, the life cycle of TGF-*β* is complex; it can exist in a variety of forms.^4^ For simplicity and following,^6^ we distinguish three forms of TGF-*β*:

- endogenous, unbound latent TGF-*β*, denoted c(x,t)
- latent TGF-*β*, bound and stored in the ECM (or, in this application, the hydrogel), denoted b(x,t)
- active TGF-*β*, either exogenous or cleaved (and activated) from the ECM, denoted a(x,t)

In its active form, TGF-*β* can bind to cell receptors on the outer membrane of MSCs and chondrocytes. We track the proportion of bound TGF-*β* receptors/cell for each cell type, and denote them by f(x,t) and g(x,t) for MSCs and chondrocytes, respectively.

Endogenous TGF-*β*, c(x,t), is secreted by chondrocytes as a large molecular complex, comprising the TGF-*β* ligand, a latency peptide which prevents interaction with cell receptors, and a protein which enables the secreted complex to bind to the hydrogel. Latent TGF-*β* diffuses freely, although the spatial extent of its diffusion is limited by the high affinity with which it binds to the hydrogel. It is assumed here that the rate of diffusion is identical in the hydrogel and the culture medium. The partial differential equation describing the evolution of c(x,t) is thus

(3) $$\frac{\partial c}{\partial t}=\underset{\mathrm{diffusion}}{\underset{︸}{D_{c}\frac{\partial^{2}c}{\partial x^{2}}}}+\underset{\begin{array}{c} \mathrm{constitutive}\;\mathrm{production}\\ \mathrm{by}\;\mathrm{chondrocytes} \end{array}}{\underset{︸}{\lambda_{3}n}}-\underset{\mathrm{binding}\;\mathrm{to}\;\mathrm{ECM}}{\underset{︸}{\lambda_{4}c}}-\underset{\mathrm{natural}\;\mathrm{decay}}{\underset{︸}{\lambda_{5}c}}$$

In equation (3), we have introduced several parameters whose definitions, along with representative values, are given in Table 2. We have also assumed that the density of the ECM/hydrogel to which the TGF-*β* binds is constant (and therefore absorbed into the parameter $\lambda_{4}$).

In order for the TGF-*β* bound to the ECM and/or hydrogel to become accessible to cells, it must be activated. Here we assume that the dominant mechanism of activation is chemical (due to extremes in pH, or interaction with proteases, thrombospondin-1, reactive oxygen species). It follows that the differential equation governing b(x,t), the concentration of hydrogel bound TGF-*β*, is

(4) $$\frac{\partial b}{\partial t}=\underset{\mathrm{binding}\;\mathrm{to}\;\mathrm{ECM}}{\underset{︸}{\lambda_{4}c}}-\underset{\mathrm{chemical}\;\mathrm{activation}}{\underset{︸}{\lambda_{6}b}}-\underset{\mathrm{natural}\;\mathrm{decay}}{\underset{︸}{\lambda_{7}b}}$$

Active TGF-*β* can bind to cells and diffuse but has a very short half-life. The equation governing the concentration of active TGF-*β*, a(x,t), is thus

(5) $$\begin{array}{l} \frac{\partial a}{\partial t}=\underset{\mathrm{diffusion}}{\underset{︸}{D_{a}\frac{\partial^{2}a}{\partial x^{2}}}}+\underset{\mathrm{chemical}\;\mathrm{activation}}{\underset{︸}{\lambda_{6}b}}-\underset{\mathrm{decay}}{\underset{︸}{\lambda_{8}a}}\\ -\underset{\mathrm{binding}\;\mathrm{to}\;\mathrm{cell}\;\mathrm{receptors}}{\underset{︸}{\left(\lambda_{m9}amF_{\mathrm{tot}}\left(1-f\right)+\lambda_{n9}anG_{\mathrm{tot}}\left(1-g\right)\right)}} \end{array}$$

In this work, we consider a simpler model of receptor-ligand dynamics than that proposed by other authors.^18^ We assume that all receptors are identical and once bound to cell surface receptors, TGF-*β* is rapidly internalised. We also assume that when an MSC differentiates any bound TGF-*β* will be bound to the newly created chondrocyte. Furthermore, we assume that the two cell types have a fixed number of receptors per cell, with the total possible mass of TGF-*β* bound to an MSC or chondrocyte denoted $F_{\mathrm{tot}}$ and $G_{\mathrm{tot}}$, respectively. The following equations describe how the fraction of bound receptors per volume changes over time for each of the two cell types

(6) $$\begin{array}{l} \frac{\partial \left(fm\right)}{\partial t}=\underset{\mathrm{binding}\;\mathrm{to}\;\mathrm{MSC}\;\mathrm{receptors}}{\underset{︸}{\lambda_{m9}am\left(1-f\right)}}-\underset{\begin{array}{c} \mathrm{internalization}\\ \left(\mathrm{MSCs}\right) \end{array}}{\underset{︸}{\lambda_{m10}fm}}\\ -\underset{\begin{array}{c} \mathrm{loss}\;\mathrm{of}\;\mathrm{bound}\;\mathrm{TGF}-\beta \\ \mathrm{due}\;\mathrm{to}\;\mathrm{MSC}\;\mathrm{differentiation} \end{array}}{\underset{︸}{\Lambda_{1}H\left(f-f_{d}\right)fm}} \end{array}$$

(7) $$\begin{array}{l} \frac{\partial \left(gn\right)}{\partial t}=\underset{\mathrm{binding}\;\mathrm{to}\;\mathrm{chondrocyte}\;\mathrm{receptors}}{\underset{︸}{\lambda_{n9}an\left(1-g\right)}}-\underset{\begin{array}{c} \mathrm{internalization}\\ \left(\mathrm{chondrocytes}\right) \end{array}}{\underset{︸}{\lambda_{n10}gn}}\\ +\underset{\begin{array}{c} \mathrm{gain}\;\mathrm{of}\;\mathrm{bound}\;\mathrm{TGF}-\beta \\ \mathrm{due}\;\mathrm{to}\;\mathrm{MSC}\;\mathrm{differentiation} \end{array}}{\underset{︸}{\Lambda_{1}H\left(f-f_{d}\right)fm}} \end{array}$$

As stated earlier, descriptions and typical values of the parameters that appear in equations (1)–(7) are given in Table 2. Where possible parameter values are taken from previous modelling studies of related biological systems, others are estimated from in vitro experiments involving TGF-*β*.

We solve equations (1)–(7) in a domain that represents a culture system comprising a cell-seeded scaffold which is bathed in culture medium (see Figure 4). The scaffold and culture medium are each assumed to be of height L. The scaffold (which contains cells) is located in the region 0<x<L and the (cell-free) culture medium is located in the region L<x<2L. No-flux boundary conditions are imposed for both diffusible species at x=0 and x=2L, so that

(8) $$\frac{\partial c}{\partial x}=\frac{\partial a}{\partial x}=0,\;\;\mathrm{on}\;x=0,2L$$

The initial conditions used for the three strategies depicted in Figure 4 are stated in terms of dimensionless variables below.

### Non-dimensionalisation, numerical solution technique

We non-dimensionalise equations (1)–(7) by scaling the cell densities and concentrations of TGF-*β* as follows (where stars denote dimensionless quantities)

(9) $$\left(a,b,c\right)=A_{0}\left(a^{*},b^{*},c^{*}\right),\;\;\left(m,n\right)=M_{0}\left(m^{*},n^{*}\right)$$

where $A_{0}=1\;\mathrm{ng}/\mathrm{mL}$ and $M_{0}=1\;\mathrm{Mio}/\mathrm{mL}$ are chosen to be in line with typical order of magnitude values for tissue engineering applications. We introduce a length scale based on the height of the scaffold and a timescale based on the timescale of interest for the experiments, given by the MSC to chondrocyte differentiation rate $\Lambda_{1}$, these are

(10) $$t=\frac{1}{\Lambda_{1}}t^{*},\;\;x=Lx^{*}$$

These scales are chosen to be L=2.4mm and $\Lambda_{1}=6\times 10^{-3}\;/\min$. Applying these scalings and dropping the stars on the dimensionless quantities, equations (1)–(7) become

(11) $$\frac{\partial m}{\partial t}=-H\left(f-f_{d}\right)m$$

(12) $$\frac{\partial n}{\partial t}=H\left(f-f_{d}\right)m$$

(13) $$\frac{\partial c}{\partial t}={\tilde{D}}_{c}\frac{\partial^{2}c}{\partial x^{2}}+{\tilde{\lambda }}_{3}n-{\tilde{\lambda }}_{4}c-{\tilde{\lambda }}_{5}c$$

(14) $$\frac{\partial b}{\partial t}={\tilde{\lambda }}_{4}c-{\tilde{\lambda }}_{6}b-{\tilde{\lambda }}_{7}b$$

(15) $$\begin{array}{l} \frac{\partial a}{\partial t}={\tilde{D}}_{a}\frac{\partial^{2}a}{\partial x^{2}}+{\tilde{\lambda }}_{6}b-{\tilde{\lambda }}_{8}a-{\tilde{\lambda }}_{m9}am\left(1-f\right)\\ \;\;-{\tilde{\lambda }}_{n9}an\left(1-g\right) \end{array}$$

(16) $$\frac{\partial \left(fm\right)}{\partial t}={\hat{\lambda }}_{m9}am\left(1-f\right)-{\tilde{\lambda }}_{m10}fm-H\left(f-f_{d}\right)fm$$

(17) $$\frac{\partial \left(gn\right)}{\partial t}={\hat{\lambda }}_{n9}an\left(1-g\right)-{\tilde{\lambda }}_{n10}gn+H\left(f-f_{d}\right)fm$$

Suitably non-dimensionalised, the boundary conditions (8) are

(18) $$\frac{\partial c}{\partial x}=\frac{\partial a}{\partial x}=0,\;\;\mathrm{on}\;x=0,2$$

Equations (11)–(17) contain a number of dimensionless parameters; they are defined in terms of the dimensional parameters in Table 3, along with estimated values based on the representative values of the dimensional parameters given in Table 2.

**Table 3.** Summary of the dimensionless parameters that appear in equations (11)–(17). Estimates of their values are based on the representative values of the dimensional parameters given in Table 2. The scaling parameters $A_{0}=1\;\;\mathrm{ng}/\mathrm{mL}$, $M_{0}=1\;\mathrm{Mio}/\mathrm{mL}$ and $L=2.4\times 10^{-3}m$.

Quantity	Definition	Value	Quantity	Definition	Value
${\tilde{\lambda }}_{3}$	$=M_{0}\lambda_{3}/(A_{0}\Lambda_{1})$	18.2	${\tilde{\lambda }}_{n9}$	$=M_{0}G_{tot}\lambda_{n9}/\Lambda_{1}$	6.23
${\tilde{\lambda }}_{4}$	$=\lambda_{4}/\Lambda_{1}$	16.7	${\hat{\lambda }}_{m9}$	$=A_{0}\lambda_{m9}/\Lambda_{1}$	10
${\tilde{\lambda }}_{5}$	$=\lambda_{5}/\Lambda_{1}$	1	${\hat{\lambda }}_{n9}$	$=A_{0}\lambda_{n9}/\Lambda_{1}$	10
${\tilde{\lambda }}_{6}$	$=\lambda_{6}/\Lambda_{1}$	10	${\tilde{\lambda }}_{m10}$	$=\lambda_{m10}/\Lambda_{1}$	41.7
${\tilde{\lambda }}_{7}$	$=\lambda_{7}/\Lambda_{1}$	1	${\tilde{\lambda }}_{n10}$	$=\lambda_{n10}/\Lambda_{1}$	41.7
${\tilde{\lambda }}_{8}$	$=\lambda_{8}/\Lambda_{1}$	43	${\tilde{D}}_{c}$	$=D_{c}/(\Lambda_{1}L^{2})$	$3.7\times 10^{-2}$
${\tilde{\lambda }}_{m9}$	$=M_{0}F_{tot}\lambda_{m9}/\Lambda_{1}s$	6.23	${\tilde{D}}_{a}$	$=D_{a}/(\Lambda_{1}L^{2})$	$3.7\times 10^{-2}$

### Initial conditions

We close equations (11)–(17), subject to boundary conditions (18), by prescribing suitable initial conditions for the three case studies of interest.

#### Case study 1

As shown in Figure 4(a), the initial cell densities for this case are

(19) m(x,0)=H(1−d−x)H(x)

(20) $$n(x,0)=n_{0}H(x-1+d)H(1-x)$$

representing a layer of MSCs of density 1 and height (1−d) below a layer of chondrocytes of (dimensionless) density $n_{0}$ and (dimensionless) height d. The system is initially devoid of any TGF-*β*, so we prescribe

(21) a(x,0)=b(x,0)=c(x,0)=f(x,0)=g(x,0)=0

Consequently, any MSC differentiation will be due to TGF-*β* constitutively produced by chondrocytes. Our simulations focus on the effect of varying $n_{0}$, the initial chondrocyte density, and d, the depth of the upper chondrocyte layer.

#### Case study 2

As shown in Figure 4(b), the initial MSC density and active TGF-*β* concentration for this scenario are

(22) m(x,0)=H(1−x)H(x)

(23) $$a(x,0)=a_{0}H(x-1)\;H(2-x)\;$$

so that initially only MSCs are present and the culture medium is filled with active TGF-*β* at concentration $a_{0}$. Chondrocytes and all other forms of TGF-*β* are absent, so that

(24) n(x,0)=b(x,0)=c(x,0)=f(x,0)=g(x,0)=0

The focus of this case study is to investigate the effect on the MSC layer of varying $a_{0}$, the initial active TGF-*β* concentration.

#### Case study 3

As shown in 4(c), the initial cell densities and active TGF-*β* concentration are now

(25) m(x,0)=H(1−d−x)H(x)

(26) $$n(x,0)=n_{0}H(x-1+d)H(1-x)$$

(27) $$a(x,0)=a_{0}H(x-1)\;H(2-x)\;$$

thus combining the layered cell seeding of case study 1 and the TGF-*β* stimulation of case study 2. All other forms of TGF-*β* are initially absent so that

(28) b(x,0)=c(x,0)=f(x,0)=g(x,0)=0

The focus here is to investigate possible synergistic effects between the two sources of TGF-*β* by varying both $a_{0}$ and $n_{0}$.

### Numerical solution procedure

Equations (11)–(17) with boundary conditions (18) are solved numerically subject to the initial conditions associated with a particular case study. To solve the governing equations, we partition the spatial domain into a grid of N equally spaced points (typically, N=200 is sufficient to provide convergence) and employ the method of lines, using central difference approximations to discretise spatial derivatives. The resulting system of time-dependent ordinary differential equations is then integrated via the Runge–Kutta (2, 3) routine ode23tb in MATLAB.

## Results/discussion

We describe below the key results from our investigations of the cell seeding and/or TGF-*β* stimulation strategies depicted in Figure 4. We also discuss briefly the dynamics that lead to chondrogenesis.

To summarise the output of our simulations, we introduce the quantity N(t) to represent the total number of MSC-derived chondrocytes per cross-sectional area at time t. This is defined as

(29) $$N(t)=\int_{0}^{1-d}n(x,t)\;dx$$

We also introduce n(x,∞) to represent the long-term chondrocyte distribution. In practice, this is taken to be the chondrocyte density at the final time in our simulations. We terminate our simulations at either $t=t_{90\%}$, the time at which 90% of the initial MSC population has differentiated into chondrocytes, or at t=20, an arbitrarily late time by which we have found the system is typically close to steady state, whichever is earlier. The choice of 90% as a cutoff here is also arbitrary but, as will be seen in the results presented herein, once 90% of the initial MSC population has been differentiated, the remaining MSCs will ultimately be driven to differentiate at later times. We define $t_{90\%}$ as

(30) $$N\left(t_{90\%}\right)=0.9\times \int_{0}^{1-d}m\left(x,0\right)\;dx$$

### Case study 1

#### MSC/chondrocyte co-culture with equal height layers

We have found that there are two possible outcomes for this strategy: if the chondrocytes are seeded at too low a density, then none of the MSCs in the lower layer differentiate; if they are seeded above a critical density of $n_{crit}\approx 0.27,$ then all of the MSCs differentiate. We note here that $n_{\mathrm{crit}}$ is subject to the choice of parameter values given in Table 3. Although reasonable in this context, the usual degree of uncertainty associated with parameter values in a mathematical model of a biological system is also associated with the predicted value of $n_{\mathrm{crit}}$.

**Figure 5.**
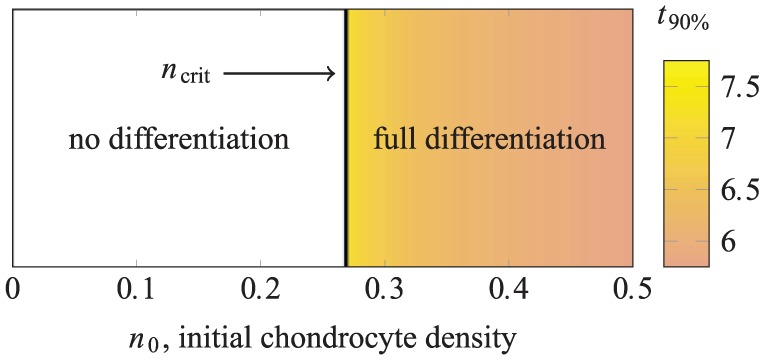
The final yield of chondrocytes in the lower layer for case study 1 as $n_{0}$, the initial density of chondrocytes in the upper layer, is varied. The vertical line indicates $n_{\mathrm{crit}}$, the critical value of initial chondrocyte density above which the constitutive production of TGF-*β* by the seeded chondrocytes is sufficient to induce differentiation of all MSCs in the lower layer. The full differentiation region is shaded to indicate $t_{90\%}$, the time after which 90% of the seeded MSCs have differentiated into chondrocytes.

Figure 5 shows how the long-term state of the system depends on $n_{0}$. The critical value $n_{\mathrm{crit}}$ is indicated with a solid line. The full differentiation region in Figure 5 is shaded to indicate the time $t_{90\%}$. This demonstrates that increasing the chondrocyte density past the critical value $n_{\mathrm{crit}}$ results in only a minimal decrease in the time taken for the majority of the MSCs to differentiate (increasing the initial density from $n_{\mathrm{crit}}$ to $n_{0}=0.5$ decreases $t_{90\%}$ by less than 5 hours in dimensional time, for instance).

This behaviour is directly analogous to that seen in a well-mixed co-culture of chondrocytes and MSCs,^6^ except that the value of $n_{\mathrm{crit}}$ here is higher than that predicted by the well-mixed model. This is to be expected since in the spatially resolved model, TGF-*β* must diffuse through the scaffold and will degrade as it does so.

Note that although the density $n_{\mathrm{crit}}$ required to induce chondrogenesis here is approximately double that required in the well-mixed case,^6^ the two approaches would require approximately the same *number* of chondrocytes to achieve full differentiation; that is, in the well-mixed case, the cells would be seeded over the full height of the construct, rather than a layer of half that height. The practical implication of this result is that spatially segregating the initial cell populations makes only minimal difference in terms of inducing chondrogenesis. As noted elsewhere, there may be other advantages to a layered arrangement (for instance, to aid in the integration of the engineered implant with subchondral bone).

#### MSC/chondrocyte co-culture where the chondrocyte layer is thin

As above, in practice, the initial number of chondrocytes, not just their density, must be considered since they are limited in supply since in the context of tissue engineering, they must be harvested from healthy tissue. A strategy that uses a relatively thin upper layer would require fewer chondrocytes than a relatively thick upper layer seeded at the same initial chondrocyte density. We suggest that such a seeding strategy may be an effective use of the available chondrocytes when they are limited in number, since they will be seeded at a higher density.

**Figure 6.**
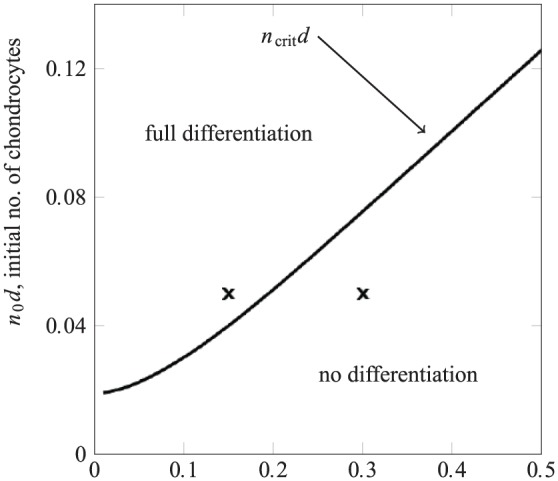
Dependence of the long term outcome on upper layer depth d and the initial number of chondrocytes seeded into the upper layer $n_{0}d$ (per cross-sectional area). The solid line divides the parameters space into regions where either no differentiation occurs or the initial MSC population is completely differentiated. The crosses indicate two seeding strategies that use the same number of initial chondrocytes with only the case represented by the leftmost cross leading to full differentiation.

The initial number of chondrocytes in the upper layer (per cross-sectional area) is given by $n_{0}d$. The effect on the long-time outcome of varying this quantity along with the layer height d is shown in Figure 6. This suggests that when only a fixed number of chondrocytes are available, then seeding these in a layer as thin as practicable (i.e. at as high density as possible) is indeed a viable and potentially efficient strategy to achieve complete differentiation of the initial MSCs.

For instance, the crosses in Figure 6 represent cases where the same number of chondrocytes are seeded at two different layer heights; the thicker layer (rightmost cross for d=0.3) does not induce chondrogenesis, while the thinner layer (leftmost cross for d=0.15) where the chondrocytes are seeded at a higher density does induce chondrogenesis.

### Case study 2

#### TGF-*β* stimulation for a pure MSC layer

We now consider a situation in which the scaffold is initially seeded with MSCs only and stimulated by exogenous TGF-*β* (see Figure 4(b)). Our simulations indicate that three long-term outcomes are possible depending on the initial concentration of exogenous TGF-*β*. For low concentrations, no cells differentiated; for moderate concentrations, some cells are differentiated near the top of the scaffold; and above a critical initial concentration $a_{\mathrm{crit}}$, the entire MSC population differentiates.

**Figure 7.**
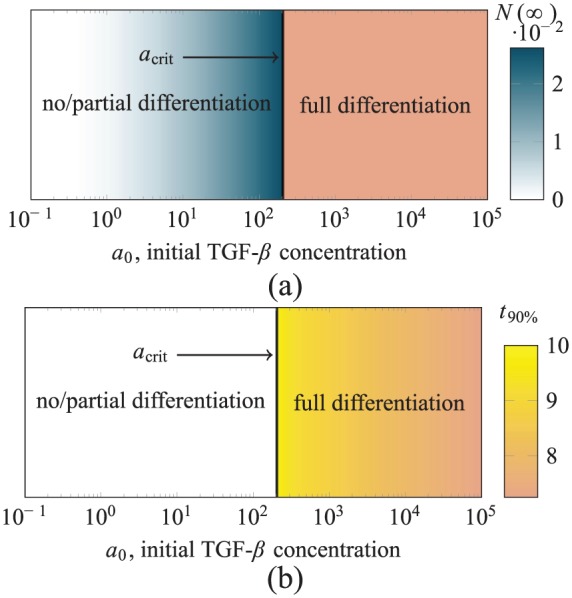
The final yield of chondrocytes for case study 2, shown over a range of $a_{0}$, the initial concentration of active TGF-*β* in the culture medium. The vertical line indicates $a_{\mathrm{crit}}$, the value of $a_{0}$ above which all MSCs are differentiated: (a) The shading of the no/partial differentiation region indicates N(∞), the number of chondrocytes produced. (b) The shading of the full differentiation region indicates $t_{90\%}$, the time by which 90% of the original MSC population has been differentiated.

The results of our simulations are summarised in Figure 7, where the shading of the upper plot indicates $N_{\infty }$ and the shading of the lower plot indicates $t_{90\%}$. Where no/partial differentiation occurs, the number of chondrocytes (per cross-sectional area) produced increases with the initial TGF-*β* concentration, up to N(∞)≈0.026, which represents 2.6% of the initial MSC population. The chondrocyte distribution in these partially differentiated long-term states consists of small number of chondrocytes in a thin layer near the top of the scaffold. The maximum chondrocyte density in this thin layer is less than $n_{\mathrm{crit}}$ (the critical value from case study 1), so no further differentiation occurs once the exogenous TGF-*β* has degraded.

Full differentiation is triggered if the initial TGF-*β* concentration $a_{0}>a_{\mathrm{crit}}\approx 203$. In terms of triggering chondrogenesis, there is no advantage in increasing the initial concentration beyond this critical value. As shown in Figure 7(b), the time $t_{90\%}$ taken to differentiate the majority of the cells slightly decreases as the initial TGF-*β* concentration is increased past $a_{\mathrm{crit}}$, although note that increasing $a_{0}$ by around two orders of magnitude only results in a small drop in $t_{90\%}$ (equivalent to approximately 6 hours in dimensional time).

The behaviour here is qualitatively similar to the analogous well-mixed case^6^ where the exogenous TGF-*β* is assumed to be evenly distributed throughout the scaffold at all times, but the corresponding initial TGF-*β* concentration required to induce chondrogenesis in the well-mixed case is an order of magnitude lower.^6^ In the layered case, the exogenous TGF-*β* must diffuse through scaffold to have an effect on the cells during which time it degrades, whereas in the well-mixed case, it was assumed to be instantaneously available at the location of the cells. The mathematical model of the present work, which includes diffusion of TGF-*β*, is a more appropriate description of experiments such as that shown in Figure 3.

### Case study 3

#### TGF-*β* stimulation for a co-culture with layers of equal height

**Figure 8.**
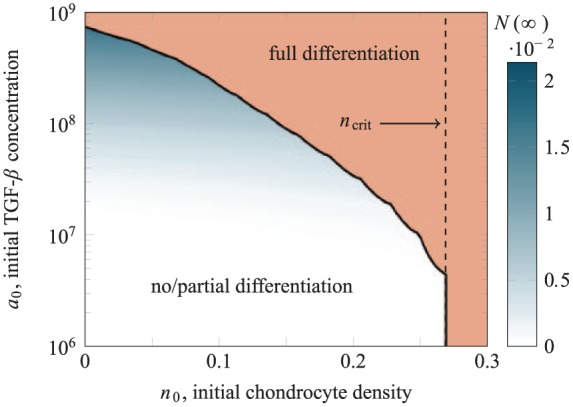
Dependence of the long-term state on $a_{0}$, the initial TGF-*β* concentration in the culture medium, and $n_{0}$, the density of chondrocytes seeded in the upper layer for layers of equal height (*d* = 0.5). The shading of the no/partial differentiation region indicates N(∞), the final number of chondrocytes in the lower layer. The uniform shading of the full differentiation region indicates that in this region, all lower layer MSCs have been differentiated. The dashed line indicates $n_{\mathrm{crit}}$, the value of $n_{0}$ above which full differentiation will occur regardless of any TGF-*β* supplementation (as in case study 1).

There are three possible outcomes for this strategy. These outcomes are summarised in Figure 8 for a range of initial TGF-*β* concentrations $a_{0}$ and initial upper layer chondrocytes densities $n_{0}$. For low initial TGF-*β* concentration and chondrocyte density less than $n_{\mathrm{crit}}$ (the value from case study 1), no MSCs are differentiated. For larger values of initial TGF-*β* concentration and initial chondrocyte density less that $n_{\mathrm{crit}}$, the cell population is partially differentiated, with N(∞) indicated by the blue shading of this region. Similar to case study 2, the chondrocyte population when partially differentiated consists of the cells initially seeded in the upper layer and a thin layer of chondrocytes that have been differentiated at the top of the lower MSC layer.

Full differentiation occurs if $n_{0}>n_{\mathrm{crit}}$ (as in case study 1) or for $n_{0}<n_{\mathrm{crit}}$ if the initial TGF-*β* concentration exceeds the critical value indicated by the curved solid line in Figure 8. This critical value is strongly dependent on $n_{0}$ and decreases by over two orders of magnitude over the range $0<n_{0}<n_{\mathrm{crit}}$. As more chondrocytes are added, the background level of constitutively produced TGF-*β* in the scaffold is raised and so less exogenous TGF-*β* is required to tip the system into a state where all MSCs eventually differentiate.

These simulations indicate that for a layered co-culture with layers of equal height, supraphysiological concentrations of TGF-*β* are required to induce chondrogenesis (physiological concentrations are less than 1 ng/mL or 1 in dimensionless concentration). Here, the upper chondrocyte layer acts as a barrier to diffusion, so very large initial concentrations of TGF-*β* must be added to the culture medium for it to have any effect; for lower initial concentrations, it will have degraded so much as to be at a negligible concentration by the time it has diffused through to the lower MSC layer. Such large concentrations of TGF-*β* are not physically realistic. For reference, the dimensionless half-life of the exogenous (active) TGF-*β* is approximately 0.016. As indicated by the value of ${\tilde{\lambda }}_{8}$ in Table 3, the timescale over which the degradation of active TGF-*β* takes place is 43 times faster than that of MSC to chondrocyte differentiation.

#### TGF-*β* stimulation for a co-culture where the chondrocyte layer is thin

**Figure 9.**
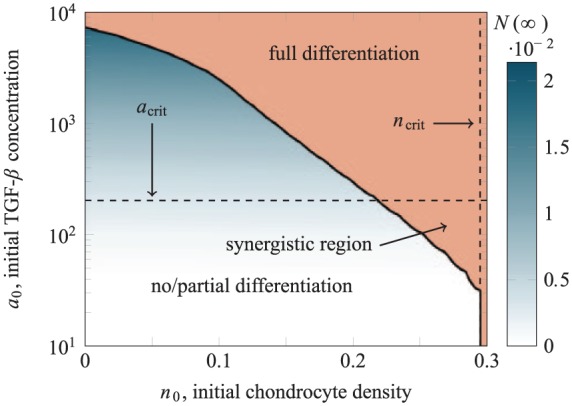
Dependence of the long-term state on $a_{0}$, the initial TGF-*β* concentration in the culture medium, and $n_{0}$, the density of chondrocytes seeded in the upper layer for a relatively thin upper layer (*d* = 0.125). The shading of the no/partial differentiation region indicates N(∞), the final number of chondrocytes in the lower layer. The uniform shading of the full differentiation region indicates that in this region, all lower layer MSCs have been differentiated. The dashed lines indicates $n_{\mathrm{crit}}$ the value of $n_{0}$ above which full differentiation will occur regardless of any TGF-*β* supplementation (as in case study 1), and $a_{\mathrm{crit}}$ the value of $a_{0}$ above which full differentiation will be driven by exogenous TGF-*β* alone.

**Figure 10.**
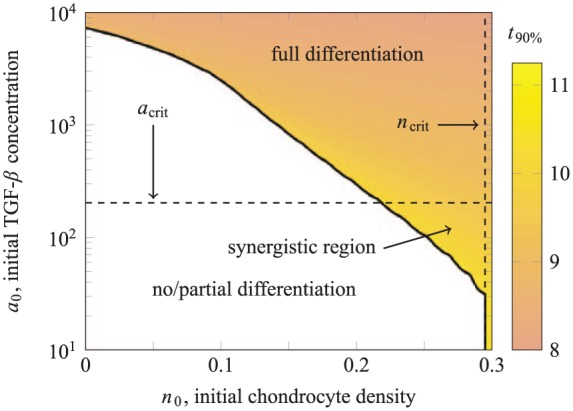
Dependence of the long-term state on $a_{0}$, the initial TGF-*β* concentration in the culture medium, and $n_{0}$, the density of chondrocytes seeded in the upper layer. The shading of the full differentiation region indicates $t_{90\%}$, the time by which 90% of the initial MSC population has differentiated into chondrocytes. The absence of shading in the no/partial differentiation region indicates that there are no choices of $n_{0}$ and $a_{0}$ in this region for which 90% of the initial MSC population will differentiate.

We hypothesise that if the relative height of the upper chondrocyte layer is reduced, it will no longer act as a barrier to diffusion of TGF-*β* from the culture medium. We have repeated our simulations for a reduced upper layer height of d=0.125 with the results summarised in Figures 9 and 10. This is qualitatively similar to the case of equal layer heights, but requires a much lower initial concentration of TGF-*β* to induce full differentiation of the MSCs.

This reveals that for a relatively thin upper layer of seeded chondrocytes, it is possible to induce chondrogenesis using fewer chondrocytes and a lower concentration of exogenous TGF-*β* than using these strategies in isolation. The critical values $n_{\mathrm{crit}}$ from case study 1 (for d=0.125) and $a_{\mathrm{crit}}$ from case study 2 are shown as dashed lines in Figures 9 and 10. A synergistic region is indicated between these dashed lines and the solid critical curve. In this region, full differentiation may be achieved using values of $n_{0}<n_{\mathrm{crit}}$ and $a_{0}<a_{\mathrm{crit}}$, representing an improvement over the situation in case studies 1 and 2 where the two sources of TGF-*β* were used individually. Here, the two sources of TGF-*β* act together in a synergistic way to differentiate the MSCs. Note that in this synergistic region, the values of $a_{0}$ and $n_{0}$ are experimentally achievable (exogenous TGF-*β* concentrations in the order of 100 ng/mL or 100 in dimensionless concentration, have been used in vitro), in contrast to the predictions for layers of equal height.

Figure 10 shows further results from our simulations, with the full differentiation region shaded for $t_{90\%}$, the time by which 90% of the initial MSC population has been differentiated into chondrocytes. This reveals that if optimising time taken to achieve differentiation is a concern, rather than just triggering chondrogenesis, then both increasing the initial chondrocyte density and increasing the initial TGF-*β* concentration beyond their critical values (represented by the solid line) can slightly reduce the time taken to achieve near complete differentiation. For instance, for $a_{0}{=10}^{2.5}\approx 316$ and $n_{0}=0.2$, there is a value of $t_{90\%}\approx \;10.0$ (or approximately 28 hours in dimensional time), but for $a_{0}{=10}^{2.5}\approx \;\;316$ and $n_{0}=0.3$, this reduces to $t_{90\%}\approx 9.1$ (approximately 25 hours in dimensional time). This is comparable to the decrease in $t_{90\%}$ seen in case study 1.

### Underlying dynamics

We conclude with a brief description of the dynamic interplay between the density of the cell populations and the various forms of TGF-*β*.

#### Case study 1

**Figure 11.**
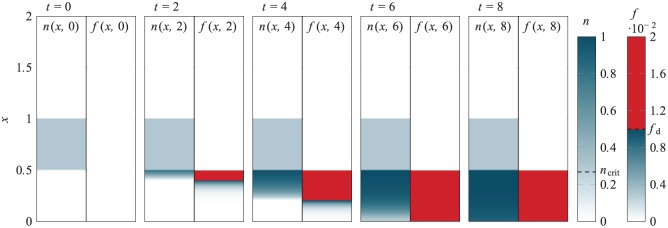
Typical simulations for case study 1 where differentiation of the MSCs in the lower layer is triggered, the exogenous TGF-*β* produced by the chondrocytes in the upper layer, shown here for an initial chondrocyte density of $n_{0}=0.3$. Chondrocyte density n(x,t) and TGF-*β* bound to MSCs f(x,t) is shown at t=0,2,4,6,8. Recall that MSC to chondrocyte differentiation is triggered when $f>f_{d}$, indicated here by red shading in the f(x,t) plots.

An example simulation for case study 1 is presented in Figure 11, showing the chondrocyte distribution n(x,t) and the amount of TGF-*β* bound to the MSCs f(x,t) at five time points. The initial chondrocyte density is $n_{0}=0.3$ which is sufficiently high that chondrogenesis is triggered and the initial population of MSCs will eventually all differentiate.

Initially, no TGF-*β* is present and so at t=0 no MSCs in the lower layer are differentiating. After a short time (t=2), the TGF-*β* constitutively produced by the upper layer chondrocytes has diffused into the lower layer in sufficient quantity (and bound to MSCs) that differentiation has been triggered at the top of the lower layer, indicated by red colouring in the f(x,t) distribution. At this early time, there is a thin layer of newly produced chondrocytes at the top of the lower layer.

These newly produced chondrocytes produce TGF-*β* to supplement that produced by the originally seeded cells. As this diffuses deeper into the lower layer, the region in which sufficient TGF-*β* is bound to the MSCs to trigger differentiation (i.e. where $f>f_{d}$) becomes thicker. Recall that $f_{d}$ is the value of f above which MSC to chondrocyte differentiation is triggered. By t=4, this region is over half the lower layer depth. As the density of the newly produced chondrocytes increases, they produce endogenous latent TGF-*β* at higher concentrations. In turn, this is stored and eventually activated, giving localised higher concentrations of active TGF-*β*. This elevated concentration of active TGF-*β* leads to more TGF-*β* being bound to MSC receptors, and where this exceeds $f>f_{d}$, the MSCs differentiate into chondrocytes.

This process continues and by t=6, there is sufficient TGF-*β* bound to MSCs to trigger differentiation throughout the lower layer, with $f>f_{d}$ for 0<x<1/2. At this time, there remains a few undifferentiated cells at the very bottom of the layer. By t=8, less than 1% of the original MSCs remain and these eventually differentiate at later times.

#### Case study 2

As summarised in Figure 7, for case study 2, the final yield of chondrocytes depends on $a_{0}$, the initial concentration of TGF-*β* added to the culture medium; for $a_{0}<a_{\mathrm{crit}}$, the initial MSC population is partially differentiated; and for $a_{0}>a_{\mathrm{crit}}$, all MSCs are differentiated.

**Figure 12.**
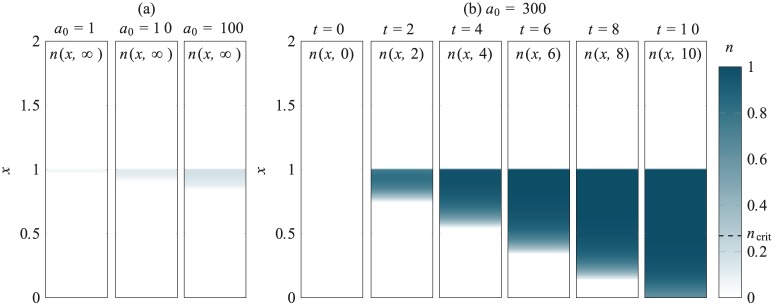
Chondrocyte densities for case study 2 (see Figure 4(b)), where MSC differentiation is induced through the initial addition of exogenous TGF-*β* of concentration $a_{0}$ in the region 1<x<2: (a) Examples of partially differentiated steady states for $a_{0}=1,\;\;10\;\;\mathrm{and}\;\;100$ and (b) for $a_{0}=300$, the initial MSC population fully differentiates, and this process is shown at six time points here. Note that in the last time step shown here, a few MSCs remain, but these will be differentiated at later times.

Three examples of partially differentiated chondrocyte distributions n(x,≈) (at steady state) are shown in Figure 12(a) for $a_{0}=1,\;\;10\;\;\mathrm{and}\;\;100$. The chondrocytes produced here are all in a thin layer near the top of the scaffold region. In these cases, the dominant source of TGF-*β* is the exogenous TGF-*β* initially added to the culture medium. This diffuses into the scaffold region in sufficient quantity, before it degrades, to trigger differentiation of MSCs near the boundary of the scaffold and culture medium resulting in a thin layer of chondrocytes. As the initial concentration of exogenous TGF-*β* is increased, the chondrocyte layer penetrates further into the scaffold and the maximum chondrocyte density increases. Crucially, this maximum density does not exceed $n_{\mathrm{crit}}$, the critical value from case study 1, and so the newly produced chondrocytes will not produce sufficient endogenous TGF-*β* to drive differentiation of remaining MSCs.

A case where enough chondrocytes are produced by the initial exogenous TGF-*β* stimulation to kick-start differentiation of the entire layer is shown in Figure 12(b) for $a_{0}=300$. Here, the chondrocytes produced at early times have a density that exceeds the threshold value $n_{\mathrm{crit}}$. Consequently, these chondrocytes produce sufficient levels of endogenous TGF-*β* to drive the differentiation of the MSCs positioned beneath them. This situation continues until all of the MSCs in the scaffold differentiate. We remark that the final time shown in Figure 12(b) is not quite steady state since a few MSCs remain undifferentiated near x=0, but these will be differentiated at a later time since $f>f_{d}$ in this region.

#### Case study 3

**Figure 13.**
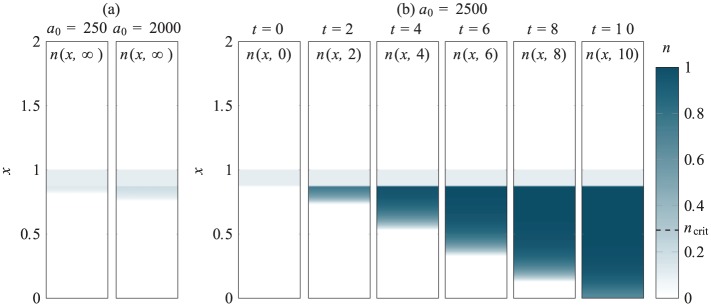
Chondrocyte densities for case study 3, where MSC differentiation is induced through the initial addition of exogenous TGF-*β* of concentration $a_{0}$ in the region 1<x<2, and chondrocytes seeded in the region 0.875<x<1 at density $n_{0}=0.1$: (a) Examples of partially differentiated steady states for $a_{0}=250\;\mathrm{and}\;2000$ and (b) for $a_{0}=2500$, the initial MSC population is driven to full differentiate, this process shown at six time steps. Note that in the last time step shown here, a few MSCs remain, but these will differentiate at later times.

The underlying dynamics of case study 3 are a combination of those seen in case studies 1 and 2. As described in Figures 8–10, the final yield of chondrocytes depends on both $a_{0}$ and $n_{0}$ (as well as the depth of the chondrocyte layer d). The exogenous TGF-*β* essentially acts to boost the TGF-*β* produced by the chondrocyte population provided it can diffuse through the chondrocyte layer in sufficient quantity before it degrades. Two examples of partially differentiated chondrocyte distributions at steady state are shown in Figure 13(a). These are both for an initial chondrocyte density of $n_{0}=0.1$ and layer height of d=0.125. For the lower concentration of $a_{0}=250$, the exogenous TGF-*β* does not diffuse into the lower layer in sufficient quantity to trigger much differentiation and only a small number of extra chondrocytes are produced at the top of the lower layer. For the higher concentration of $a_{0}=2000$, the exogenous TGF-*β* diffuses into the upper layer in to a greater extent, but similarly this only results in the production of a small number of chondrocyte near the top of the layer, albeit at a higher density and with more penetration into the layer than the lower concentration. Although these newly produced chondrocytes are an added source of endogenous TGF-*β*, they do no produce enough to trigger differentiation of the cells in the layer below.

As shown in Figure 13(b), when $a_{0}=2500$, the initial concentration of exogenous TGF-*β* is high enough to trigger differentiation of all the MSCs. Here the chondrocytes produced at early times near the top of the lower layer, together with the seeded chondrocytes, produce sufficient TGF-*β* to kickstart differentiation of all the MSCs in the lower layer. The differentiation front progresses through the depth of the layer in a similar manner to case study 2 so that by t=10, only a few MSCs remain undifferentiated at the very bottom of the scaffold. Since $f>f_{d}$ throughout the lower layer (due to the new produced chondrocytes), these remaining cells will ultimately differentiate at a later time.

## Conclusion

We have developed a mathematical model to simulate TGF-*β* mediated differentiation of MSCs into chondrocytes in tissue engineering scaffolds. This model was used to investigate a variety of experimental strategies, including layered co-cultures of MSCs and chondrocytes. The model accounts for spatial variation in the distribution of the cell populations and allows for the diffusive transport of latent and active TGF-*β*. Our extensive simulations provide insight into the efficacy of strategies currently used to engineer cartilage in vitro and suggest directions for further experimental studies.

In case study 1, we considered a layered scaffold where differentiation of the MSCs in the lower layer was driven by the endogenous production of TGF-*β* by chondrocytes seeded in the upper layer. Our key findings were that

- The initial density of chondrocytes must be above a threshold value $n_{\mathrm{crit}}$ to trigger chondrogenesis and
- Where a fixed number of chondrocytes is available, an efficient strategy is to seed them in as thin a layer as practicable.

In case study 2. we considered a scaffold seeded with only MSCs and differentiation driven by exogenous TGF-*β* added to the culture medium above the scaffold. Our key findings were that

- The initial concentration of exogenous TGF-*β* must be above a threshold value $a_{\mathrm{crit}}$ to trigger the complete chondrogenesis of the seeded MSCs and
- Adding exogenous TGF-*β* in excess of this threshold concentration results in only a small decrease in the time taken to differentiate the seeded MSCs.

Case studies 1 and 2 serve as a foundation to examine the strategy of case study 3, a layered co-culture of MSCs lying above chondrocytes, supplemented with exogenous TGF-*β*. Our key findings were that

- For layers of equal height, the exogenous TGF-*β* did not adequately diffuse through the upper chondrocyte layer to supplement that produced endogenously, and therefore had no effect in terms of promoting chondrogenesis and
- When the upper layer depth is reduced, synergistic effects are possible where chondrogenesis may be triggered using a lower concentration of exogenous TGF-*β* and a lower density of seeded chondrocytes than would be required if these sources of TGF-*β* were used in isolation.

Future work may include experimental testing of scaffolds with layered cell seeding and a relatively thin chondrocyte-seeded upper layer. The mathematical model may be extended to consider scaffolds in three spatial dimensions. Other novel protocols and cell seeding strategies not previously tested experimentally may also be considered; for example, scaffolds with more than two layers which could be seeded with cell types at different densities. We could also consider the mechanism behind mechanically induced chondrogenesis by modelling TGF-*β* activation via integrin mediated mechanotransduction^3^ and the subsequent remodelling of the mechanical environment of the scaffold as ECM components are produced by the chondrocytes.

Our simulations suggest improvements to existing experimental protocols. In particular, where layered cell seeding is used with chondrocytes lying above MSCs, there is an advantage in seeding the available chondrocytes in as thin as practicable regardless of exogenous TGF-*β* supplementation. In a strategy where no exogenous TGF-*β* is added, then for a fixed number of chondrocytes, the density will be higher if the layer is thinner, thus promoting chondrogenesis in a more efficient manner. Similarly, if layered cell seeding is used in combination with exogenous TGF-*β* stimulation, the upper layer should be as thin as practicable to permit diffusion of TGF-*β*, which in turn triggers differentiation of the seeded MSCs.
